# Flying, phones and flu: Anonymized call records suggest that Keflavik International Airport introduced pandemic H1N1 into Iceland in 2009

**DOI:** 10.1111/irv.12690

**Published:** 2019-11-09

**Authors:** Nishant Kishore, Rebecca Mitchell, Timothy L. Lash, Carrie Reed, Leon Danon, Guðrún Sigmundsdóttir, Ymir Vigfusson

**Affiliations:** ^1^ Department of Epidemiology T.H. Chan School of Public Health Harvard University Boston MA USA; ^2^ Department of Computer Science Emory University Atlanta GA USA; ^3^ Nell Hodgson Woodruff School of Nursing Emory University Atlanta GA USA; ^4^ Department of Epidemiology Rollins School of Public Health Atlanta GA USA; ^5^ Epidemiology and Prevention Branch Influenza Division National Center for Immunization and Respiratory Diseases Centers for Disease Control and Prevention Atlanta GA USA; ^6^ College of Engineering, Mathematics and Physical Sciences University of Exeter Exeter UK; ^7^ Alan Turing Institute, British Library London UK; ^8^ Centre for Health Security and Communicable Disease Center Control Directorate of Health of Iceland Reykjavík Iceland; ^9^ School of Computer Science Reykjavík University Reykjavík Iceland

**Keywords:** big data, call detail records, case‐control studies, Iceland, influenza, pandemics

## Abstract

**Background:**

Data collected by mobile devices can augment surveillance of epidemics in real time. However, methods and evidence for the integration of these data into modern surveillance systems are sparse. We linked call detail records (CDR) with an influenza‐like illness (ILI) registry and evaluated the role that Icelandic international travellers played in the introduction and propagation of influenza A/H1N1pdm09 virus in Iceland through the course of the 2009 pandemic.

**Methods:**

This nested case‐control study compared odds of exposure to Keflavik International Airport among cases and matched controls producing longitudinal two‐week matched odds ratios (mORs) from August to December 2009. We further evaluated rates of ILI among 1st‐ and 2nd‐degree phone connections of cases compared to their matched controls.

**Results:**

The mOR was elevated in the initial stages of the epidemic from 7 August until 21 August (mOR = 2.53; 95% confidence interval (CI) = 1.35, 4.78). During the two‐week period from 17 August through 31 August, we calculated the two‐week incidence density ratio of ILI among 1st‐degree connections to be 2.96 (95% CI: 1.43, 5.84).

**Conclusions:**

Exposure to Keflavik International Airport increased the risk of incident ILI diagnoses during the initial stages of the epidemic. Using these methods for other regions of Iceland, we evaluated the geographic spread of ILI over the course of the epidemic. Our methods were validated through similar evaluation of a domestic airport. The techniques described in this study can be used for hypothesis‐driven evaluations of locations and behaviours during an epidemic and their associations with health outcomes.

## 1 INTRODUCTION

Epidemiologic surveillance systems conventionally rely on passive reporting from healthcare providers and from active investigation in the field. The avalanche of data collected in our increasingly digital world provides an opportunity to incorporate user‐generated information into these surveillance systems. In particular, the spatial and temporal data collected routinely by mobile devices, which are carried by a large and growing proportion of the world's population, can provide a granular understanding of disease dynamics in real time that vastly exceeds what can be delivered by conventional surveillance systems.^1^, ^2^, ^3^, ^4^

The mechanisms of international propagation of communicable diseases remain an important topic in the study of pandemics. Influenza A/H1N1pdm09 virus was first detected in the United States in April 2009 and resulted in approximately 200 000 laboratory‐confirmed deaths worldwide over the span of the first year of virus circulation.^5^, ^6^, ^7^, ^8^, ^9^ The pandemic spread outwards from urban travel hubs and took advantage of the relatively innocuous initial symptoms of an infectious carrier, with international travellers playing a key role in transmission between continents.^10^, ^11^, ^12^, ^13^, ^14^ The highly connected air travel network and international travellers facilitated the spread of the disease, with the number of cases rapidly increasing after the initial introduction of the virus in each country.^5^, ^15^, ^16^

The ability to monitor the interaction between travel patterns and disease spread remains both an important goal and a difficult challenge for public health surveillance. The technical limitations are exacerbated by the lack of readily deployable analytic pipelines that can be scaled to analyse country‐level surveillance data in epidemic settings.^6^, ^17^ An ideal, modern, resource‐efficient surveillance system would incorporate data streams of disease detection with granular records of spatial and temporal dynamics while preserving the anonymity of individuals.

We focus our attention on call detail records (CDR), which are metadata collected by mobile network operators (MNOs) for billing purposes. CDR are an attractive data source for epidemiological surveillance. First, mobile phone use is ubiquitous, with even low‐to‐middle‐income countries having 95% penetration of mobile phones, compared, for example, to only 40% of the population having access to the Internet.^18^ Second, CDR provide relatively granular information about where and when their users have travelled, data that can be rapidly anonymized and aggregated by the MNO into an analytic data set for epidemiological analysis. Finally, as we show below, CDR can be joined with health data without an undue burden on resource‐limited health systems while preserving individual privacy.

We study the introduction of pandemic influenza A/H1N1pdm09 virus to Iceland in 2009; an isolated island with centralized national health records including influenza‐like illness (ILI) diagnoses, near‐ubiquitous mobile phone use and one likely port of entry: Keflavik International Airport.^19^, ^20^, ^21^, ^22^ We obtained anonymized CDR metadata from one of Iceland's largest mobile network operators during the period of the first wave of pandemic H1N1pdm09 virus in Iceland (August‐December), linked with health records provided by the Chief Epidemiologist at the Centre for Health Security and Communicable Disease Control of the Directorate of Health in Iceland (CHS‐CDC). Using CDR, we evaluate the role that Icelandic international travellers played in the introduction and propagation of H1N1pdm09 virus in Iceland by quantifying the association between international travel and incident ILI cases through the course of the epidemic. We show that CDR can be used as a proxy for physical proximity, allowing for the analysis of transmission dynamics of H1N1pdm09 virus within social networks over the course of the epidemic. These investigations demonstrate the relevance of CDR to epidemiologic research.

## 2 METHODS

### 2.1 Study population and design

We performed a nested case‐control study of Icelanders diagnosed with an ILI between January 2009 and March 2010. The source population consisted of 342 369 distinct phone numbers belonging to Icelanders who owned and used a personal mobile phone operated by the largest MNO in the country during the study period. The CHS‐CDC recorded 9887 incident ILI cases during the study period, 4347 of which were among clients of the sample MNO. In accordance with privacy standards, no demographic or personal identifiable information, such as age or gender, was linked to this data set. In 2009, this record likely contained mainly adults and teenagers; children too young to own a phone would not appear in the data set. We received approval #VSNb2010050012 from the National Bioethics Committee of Iceland to conduct the study as non‐human subjects research.

### 2.2 Data sets—MNO call detail records

Our CDR provide anonymized mobile phone use data from 30% to 40% of the Icelandic population over the course of 18 months, including the 6 months at the peak of H1N1pdm09 virus circulation in Iceland from August through December of 2009. The CDR database included 1 517 276 930 calls, texts and data interactions made by 342 369 unique phone numbers from 483 mobile phone tower locations during the study period. It contained encrypted mobile numbers for senders and receivers of the interaction, GPS coordinates of the tower used by the customer, timestamp of interaction, type of interaction (incoming or outgoing call or text message) and length of the interaction. The data were logged automatically and provided directly from the MNO. The data in 2009 provided a representative sample of between a third and half of the mobile phone users in Iceland.

### 2.3 Data sets—CHS‐CDC ILI diagnoses

When Icelandic physicians suspected influenza or influenza was laboratory confirmed, they were required to enter the ICD‐10 codes for ILI and confirmed influenza diagnoses in electronic patient journals. These codes were automatically selected from the patient records and reported within 24 hours via a closed electronic network to the CHS‐CDC comprising all healthcare centres and emergency rooms at hospitals in Iceland.^23^ The database of ILI diagnoses for this study contained a record of all individuals diagnosed with an ILI who were in the CDR database and included their date of diagnosis and encrypted national identification numbers (ENIN), comprising approximately half of all ILI cases.

### 2.4 Data acquisition and cleaning

The MNO provided the CDC‐CHS with a list of encrypted mobile numbers and national identification numbers. The CDC‐CHS then linked ILI information using the national identification numbers and provided the researchers with a database of encrypted mobile numbers and diagnosis information. The exchange protocol ensures that the MNO does not learn diagnosis information, and that the researchers learn neither the true mobile phone numbers nor the true national identification numbers of the individuals in the data.

A mobile phone number could be used by multiple individuals over time, and a single individual could pay for multiple mobile phone subscriptions. We restricted the data to ENIN that had a single mobile phone subscription during the study period or had multiple mobile phone subscriptions, but did not make calls between those numbers, overlapping calls or successive calls within a 5‐minute time span from towers far apart, defined by at least 10‐km great‐circle distance and in non‐adjacent Voronoi cells over tower locations.^24^ We excluded towers known to be mobile, such as towers mounted on cruise ships.

We used the Bandicoot framework to generate user‐level metrics from the larger CDR data set and further restricted the sample to individuals for whom we were able to impute home tower locations.^25^ Home towers were estimated based on identification of the tower through which most of the user's interactions were routed between the hours of 7 pm and 7 am

### 2.5 Variables

We defined cases as exposed if they had a mobile phone interaction routed through one of six mobile towers exclusively serving the Keflavik International Airport in the 4 days before their ILI diagnosis, including the day of diagnosis. Up to 20 at‐risk controls were matched to cases on home tower and sampled at random from those at risk for ILI at the time of diagnosis for the case. All cases had at least one matched control. They were evaluated similarly to exposed cases using the ILI diagnosis date of the matched case and using call records in the two weeks before and after ILI diagnosis.

### 2.6 Exposure analysis

We restricted our analysis to a continuous five‐month period from the start of August until the end of December of 2009. This period includes 92% of cases in our ILI diagnosis data set. We computed matched odds ratios (mORs) associating exposure to Keflavik International airport with ILI diagnosis for a moving two‐week window of time, resulting in a longitudinal two‐week odds of exposure and its 95% confidence interval for each day in our evaluation period. We selected a two‐week window to ensure that we captured both the incubation and infectious period of H1N1pdm09. We compared this to a two‐week period during the peak of the epidemic from 6 October through 24 October 2009. The temporal segmentation allows for the instantaneous mOR at one time to be compared to another effectively comparing relative risks.

### 2.7 Positive inferential control locations

We selected the Landspítali University Hospital, the largest hospital in Reykjavík, and the domestic airport in Akureyri, a remote city in northern Iceland, as positive inferential control locations. These selections rest on the validity of the following assumptions: we expect to see increase in the odds of exposure to the hospital, since cases would likely concentrate there in the preliminary stages of the epidemic; and we expect to see no association at Akureyri domestic airport during the initial period of the epidemic, but expect to find an increase in odds of exposure later in the epidemic when H1N1pdm09 virus had spread throughout the island.

### 2.8 Negative inferential control locations

There are several domestic airports in Iceland that provide regular passenger and cargo transport across Iceland and serve as backup ports of entry for international entry. However, they were rarely used for international travel in 2009, with fewer than 0.5% of all international flights landing outside of Keflavik.^22^, ^26^ We evaluated the domestic airport at Akureyri, the second largest by traffic volume after Reykjavik Domestic Airport, as negative control during the preliminary stages of the epidemic. The selection assumes that most international travellers do not enter this airport, but that the airport shares characteristics as a point of domestic travel with the international airport. Null associations at the domestic airport during the preliminary stages of the epidemic suggest that elevated associations near the international airport at the preliminary stages of the epidemic arise from introduction of H1N1pdm09 virus at this primary point of international entry.

### 2.9 Social network analysis

We define an ego network centred on individual *A* as follows. With vertices representing individuals, we draw an arc from individual *i* and *j* if *j* is one of *i's* 30 most frequent contacts in the two months before and after the date of *A*’s ILI diagnosis (or, if *A* is a control, the date of ILI diagnosis for its matched case). Connections of degree *k* of *A* are defined as the individuals in the *k*‐th level (*k* steps from the root, *A*) of the directed tree (arborescence) spanning the ego network centred on *A*. For example, connections of degree 2 are the set of contacts of *A*’s contacts who are not *A*’s direct contacts.

We evaluated the 10‐day rate of ILI of 1st‐degree connections after the diagnosis date of each case compared to 1st‐degree connections of each corresponding matched control. We used the same framework to evaluate the 2nd‐degree connections. We estimated the rate ratio of ILI diagnosis among 1st‐ and 2nd‐degree connections of cases compared with those of matched controls at the early stages of the epidemic against the same measure during the peak of the epidemic. All analyses were conducted using R statistical software.^27^

## 3 RESULTS

### 3.1 Descriptive data

We extracted 195 481 individual records from the ~1.5 billion call and text data read into Bandicoot. Restrictions described above narrowed the final data set to 114 293 individual records, which contained 2915 ILI cases (Table 1). These represented 62% of CDR linked ILI cases during the study period.

**Table 1:** Original and derived data sets used in our study

Description	Total	% Icelandic population^b^
Distinct MNO IDs in CDR corpus February 2009‐June 2012	342 369	107.2%
of which mobile subscription data were available	218 879	68.5%
of which subscriber had a single ENIN	171 406	53.7%
of which active between August‐December 2009^a^	114 293	35.8%
CDC‐CHS ILI records in 2009	9887	3.1%
of which the ENIN matched any MNO ID	4347	1.4%
of which single ENIN MNO ID, active Aug‐Dec 2009	2915	0.9%

Note

Data were processed into a final analytic data set from the raw records for analysis.

Abbreviations: CDR, call detail records; ENIN, encrypted Icelandic national identification number on mobile subscriptions; MNO ID, identification number of individual mobile subscriptions.

a All cases and matched controls came from this final population with active call records.

b Shown for relative comparison only, MNO IDs do not uniquely correspond to individuals.

### 3.2 Evaluation of exposure

**Figure 1:**
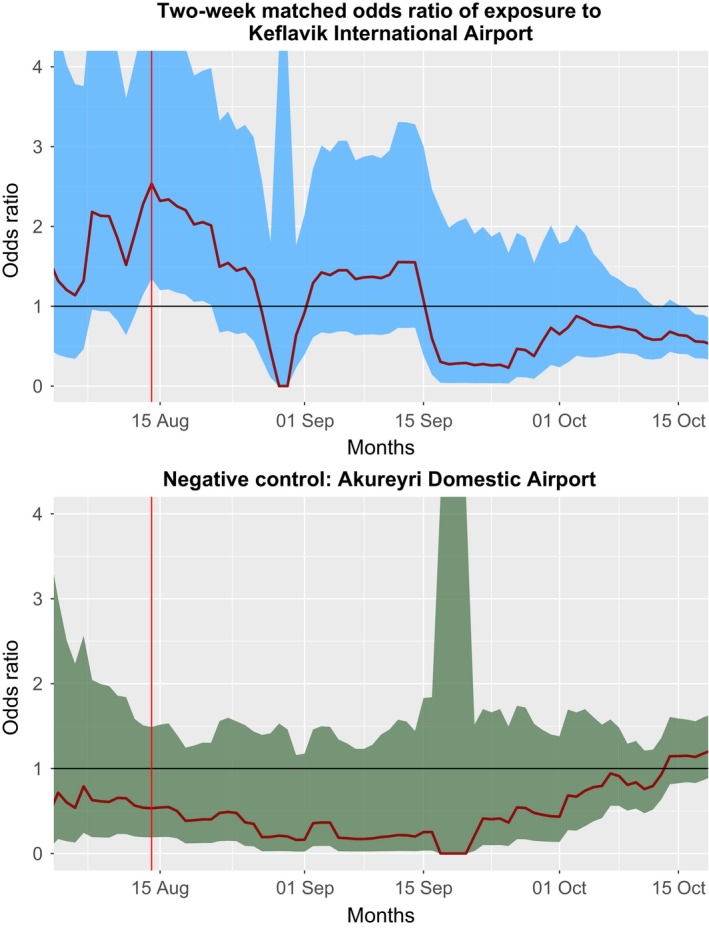
This two‐week moving window of matched odds ratios shows an increased odds of exposure to Keflavik International Airport during the initial stages of the epidemic in August of 2009. Negative controls show no similar signal

The 2‐week mORs for individuals who were exposed to the international airport in the 4 days before ILI diagnosis were calculated for every date in the study period and found to be elevated in the early stages of the epidemic (from 7 August until 21 August, mOR = 2.53; 95% CI: 1.35, 4.78) (Figure 1, Table 2).

**Table 2:** Associations between exposures of interest and subsequent ILI diagnosis with controls matched on home tower location

	Period of interest^a^	Initial stages of the epidemic^b^	Two‐week period of high risk in initial stages^c^	Comparison two‐week period in epidemic peak^d^
Primary exposures of interest—mOR [95% CI]				
Keflavik International Airport	0.88 [0.39, 3.57]	1.51 [0.71, 3.6]	2.53 [1.35, 4.78]	0.68 [0.43, 1.08]
Landspítali Hospital in Reykjavik	0.71 [0.39, 3.33]	0.96 [0.25, 4.64]	0.92 [0.22, 3.84]	1.12 [0.73, 1.72]
Negative control—mOR [95% CI]				
Akureyri Domestic Airport	0.87 [0.41, 4.39]	0.39 [0.10, 1.67]	0.53 [0.19, 1.49]	1.14 [0.82, 1.61]

Note

There is an increase in the odds of exposure to Keflavik International Airport in the initial period of the epidemic. Negative controls show a null association in the same window. Landspítali Hospital, in Reykjavik, shows an increased odds ratio near the peak of the epidemic.

Abbreviation: mOR, matched odds ratio.

a August 2009 through December 2009.

b 1 August through 15 September 2009.

c 7 August through 21 August 2009.

d 7 October through 23 October 2009.

### 3.3 Positive inferential controls

**Figure 2:**
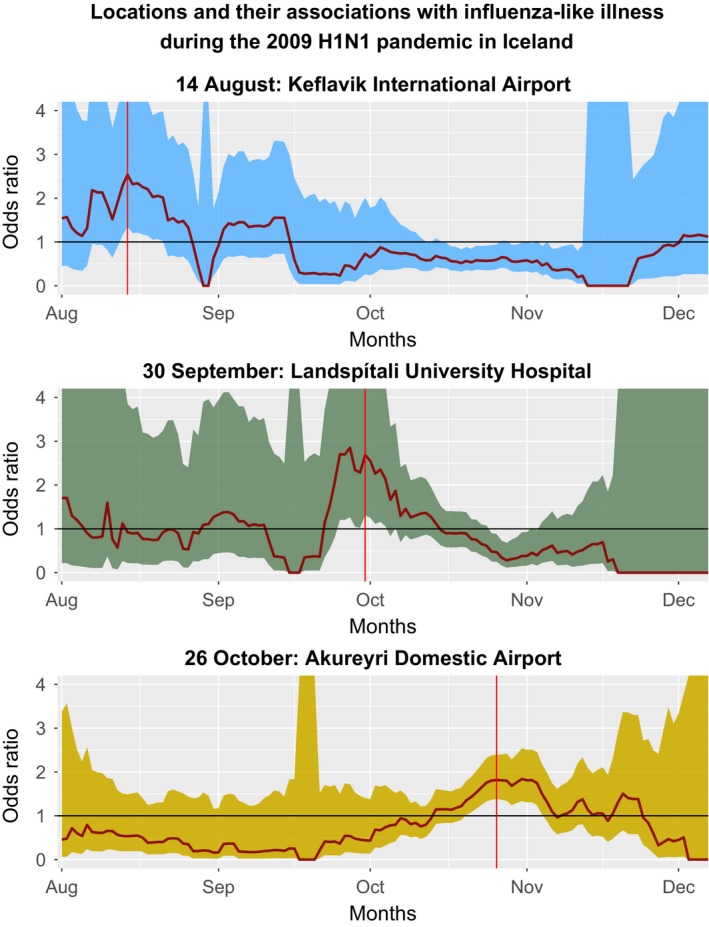
This analysis can be generalized to evaluate any spatial segment, defined by a mobile phone tower, and its role in the dynamics of influenza‐like illness during the H1N1 pandemic. We see that the increase in odds ratio is observed first at Keflavik International Airport during the initial stages of the epidemic in August, then at Landspítali Hospital during the peak of the epidemic in September and finally in the remote town of Akureyri during the end of the epidemic in November

**Figure 3:**
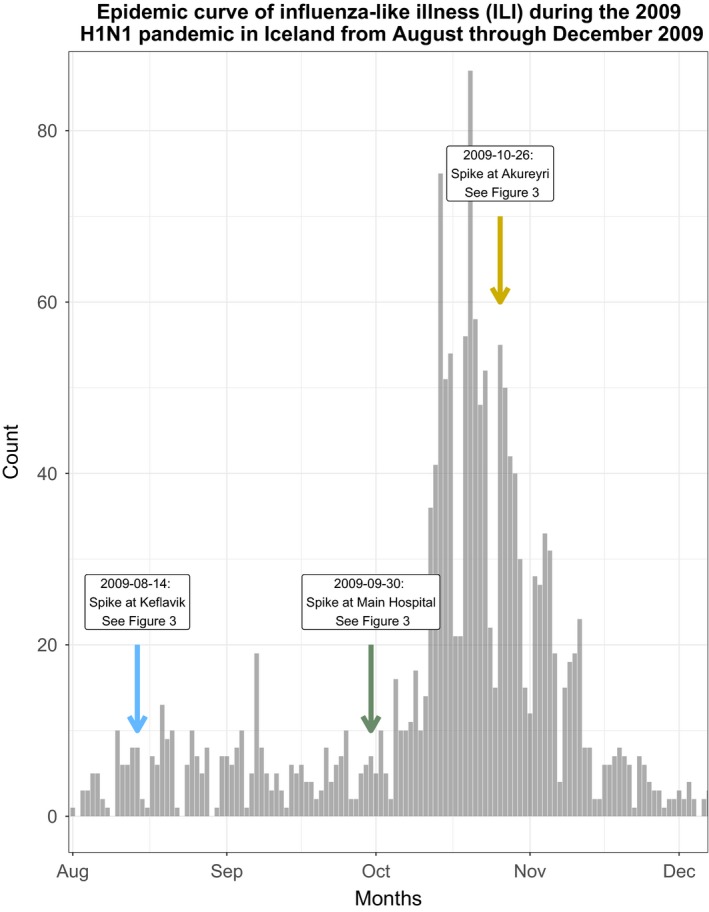
The epidemic curve of influenza‐like illness (ILI) diagnoses in Iceland from August through December 2009. The epidemic begins in late August with a peak number of cases in September and a decrease in the number of cases till December

We evaluated the major hospital in Reykjavik along with the domestic airport of Akureyri as positive inferential controls. At the hospital, we detected an increase in mOR in the two‐week period before the increase in the number of epidemic cases from 23 September until 7 October with a mOR of 2.69 (95% CI: 1.48, 5.34) (Figure 1). At Akureyri, we detected an increase in mOR in the two‐week period just after the peak of the epidemic from 19 October until the 31st with a mOR of 1.82 (95% CI: 1.38, 2.39). Chronologically, the odds of exposure spiked first at the international airport, followed by at the major hospital immediately before the increase in cases in the epidemic curve, and ending with the peak in Akureyri during the epidemic peak (Figures 2, 3).

### 3.4 Negative inferential controls

Reykjavík airport serviced the largest number of domestic flights with scheduled services in Iceland in 2009. However, mobile phone towers in the area did not receive a volume of data 22 comparable to other towers during the evaluation period. The airport also serviced small private international flights. Due to the lack of data at this location and inclusion of non‐domestic flights, we evaluated a popular domestic airport as negative controls during August‐October. As expected, the negative control showed a null or protective mOR in the early stages of the epidemic (Figure 1).

### 3.5 Social network analysis

**Figure 4:**
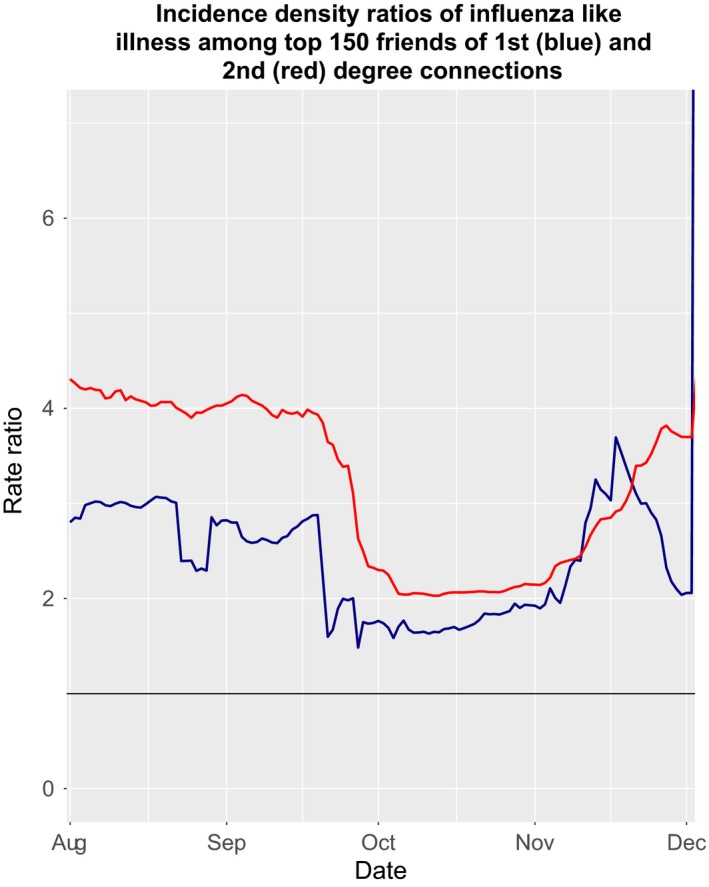
First‐degree connections have an increased incidence rate of influenza‐like illness (ILI) diagnosis during the initial stages of the epidemic. Second‐degree connections have a much higher rate initially as the population is still composed of susceptibles and the size of the second‐degree network is larger than the first. Both incidence density ratios (IDR) decrease through the course of the epidemic as the number of susceptibles in the population decreases. The IDRs cross on 7 November just after the peak of the epidemic as susceptibles no longer make up the largest portion of the population

We conducted an analysis of 10‐day rate of ILI within 1st‐degree connections among cases and controls during the initial period of the epidemic and during the epidemic peak, in both exposure levels. During the initial period of the epidemic, 1st‐degree connections of cases had an ILI rate that was 2.96 times greater than 1st‐degree connections of controls (95% CI: 1.43, 5.84) (Table 3, Figure 4, Figure S1). In contrast, during the epidemic peak, 1st‐degree connections of cases had an ILI rate 1.68 (95% CI: 1.33, 2.06) times greater than 1st‐degree connections of controls. Similarly, 2nd‐degree connections of cases had an ILI rate that was 4.09 times greater than 2nd‐degree connections of controls (95% CI: 3.81, 4.41) during the initial period of the epidemic, compared with during the epidemic peak, when 2nd‐degree connections of cases had an ILI rate that was 2.05 times greater than 2nd‐degree connections of controls (95% CI: 2.01, 2.08).

**Table 3:** The analysis of two‐week moving incidence density ratio (IDR) shows that call detail records (CDR) contact networks behave similarly to real‐world physical contact networks

Time periods of interest—IDR [95% CI]	Two‐week period of high risk in Initial Stages^a^	Comparison two‐week period in Epidemic Peak^b^
1st‐degree connections	2.96 [1.43, 5.84]	1.68 [1.33, 2.06]
2nd‐degree connections	4.09 [3.81, 4.41]	2.05 [2.01, 2.08]

Note

First‐degree connections have an increased incidence rate of influenza‐like illness (ILI) diagnosis during the initial stages of the epidemic. Second‐degree connections have a much higher rate as the population is still composed of susceptibles and the size of the second‐degree network is larger than the first. Both these increases in IDR decrease through the course of the epidemic as the number of susceptibles in the population decreases.

a 7 August through 11 August 2009.

b 7 October through 23 October 2009.

## 4 CONCLUSIONS

### 4.1 Primary findings

Our study evaluated the role that international travellers played in the introduction and propagation of pandemic influenza A/H1N1pdm09 virus in Iceland. We found that there was an association between exposure to Keflavik International Airport and incident ILI diagnoses during the initial stages of the epidemic [14 August 2‐week mOR: 2.53 (95% CI: 1.35, 4.78)].

### 4.2 Negative controls

Visiting a domestic airport was associated with no significant change in ILI risk, where visiting the Keflavik International Airport was associated with an increased ILI risk, especially early in the epidemic.

### 4.3 Secondary findings

We evaluated the rates of ILI among 1st‐degree connections of cases compared with 1st‐degree connections of controls. We expected the comparative incidence density ratio (IDR) to be high during the initial stages of the epidemic and the data aggregated from the call detail records confirmed our belief (Figure 4). Our data show that there was a higher rate of transmission to 1st‐degree connections earlier in the epidemic. However, even during the peak of the epidemic in October, when there was a generalized epidemic in the population, 1st‐degree connections of individuals diagnosed with an ILI got sick at a rate 1.67 times higher rate than the 1st‐degree connections of their matched controls. We found that IDR were higher for 2nd degree than 1st‐degree connections of cases as 2nd‐degree connections are still composed of susceptibles and have a relative size that is much larger than 1st‐degree networks. The elevated IDR in both networks decrease through the course of the epidemic as the number of susceptibles in the population decreases. The 1st‐ and 2nd‐degree rate ratios cross in 7 November as the epidemic peak is crossed, the at‐risk population decreases, and 2nd‐degree connections begin to get sick at higher rates in the control social network, mimicking real‐world contact networks.

### 4.4 Locations and their roles in an epidemic

We saw temporally local amplification of odds of ILI associated with specific regions of interest. The utility of this type of evaluation is especially important in the progression of an epidemic. For example, in Gabon, H1N1pdm09 virus propagated in urban centres during the early stages of the epidemic before expanding through transport networks to rural areas.^14^ Such propagation is demonstrated in our data through the evaluation of our positive controls. As there is only one major point of entry into Iceland, we expect an epidemic to be introduced there first, followed by transmission in areas where sick patients congregate, such as a major hospital, and finally a remote city during the peak of the epidemic in the general population. As expected, we saw clear spikes in 2‐week odds of exposure to geographic locations, moving temporally from 7 August through 14 November, and moving from the Keflavik International Airport to Landspítali University Hospital in Reykjavík and finally to the remote city of Akureyri after the epidemic peak.

### 4.5 Limitations

Call detail records for this study were captured in 2009 and 2010 when most billing activity consisted of calls and texts rather than data transactions. Therefore, individual records were dependent on users interacting with their mobile device. We were unable to discern a user's location during periods when they did not make calls, send texts or use mobile data. In contrast, current smartphones generate large records of mobile Internet data transactions and regularly request and receive updates regardless of user interaction, which would provide even more detailed location data.

Electronic health records and data privacy laws also vary significantly by country. This limits the direct application of these methods in all circumstances. However, the results of our study highlight the need for greater public‐private cooperation in the inclusion of CDR data into regular epidemiologic surveillance.

The degree of uncertainty around the mOR estimates in the early stages of the pandemic is driven by a relatively small number of cases in that time period. Future studies might incorporate larger user bases; however, diseases with relatively low baseline incidences are, by definition, sparse in data and will inherently show greater variation in the time period before and after an epidemic.

Our study was limited to the use of data from individuals who had an ENIN, in this case all Icelandic residents. While foreign visitors may have played a role in the propagation of the epidemic, they would be difficult to locate in the national electronic disease surveillance system without a local ENIN. Furthermore, it would be challenging to define a comparable control for this group as their limited CDR information may be too sparse to define variables such as “home tower.”

Finally, the use of ILI diagnosis found in the electronic medical records may not be a perfect match for a true influenza infection. A smaller validation study might be conducted in future research to examine the potential extent of misclassification caused by the use of electronic health records.

### 4.6 Future analyses

The results of this study highlight the relevance of call detail records to epidemiologic practice. Since the collection of these data in 2009, the global number of mobile phone subscriptions has risen from 68 to 103.5 per 100 inhabitants,^18^ the world population has flocked to urban centres,^28^ and nearly 2 billion new smart phone users have been registered with 6 billion projected by 2020.^29^ Modern CDR include considerably more data transfer information, allowing for more robust analyses of location and fewer threats to validity from misclassification. These data sources can provide an opportunity for both retrospective research and prospective disease surveillance. Through greater collaboration with both mobile network operators and health officials, the techniques described in this study can be used for hypothesis‐driven evaluations of locations and social networks in relation to communicable diseases during an epidemic.
