# Supplementary material for: Flying, phones and flu: Anonymized call records suggest that Keflavik International Airport introduced pandemic H1N1 into Iceland in 2009
Source: Influenza Other Respir Viruses. 2019 Nov 9;14(1):37–45. doi: 10.1111/irv.12690 (PMC6928030; doi:10.1111/irv.12690)

Figure 4: Incidence density ratios of influenza like illness among top 150 friends of 1st (blue) and 2nd (red) degree connections

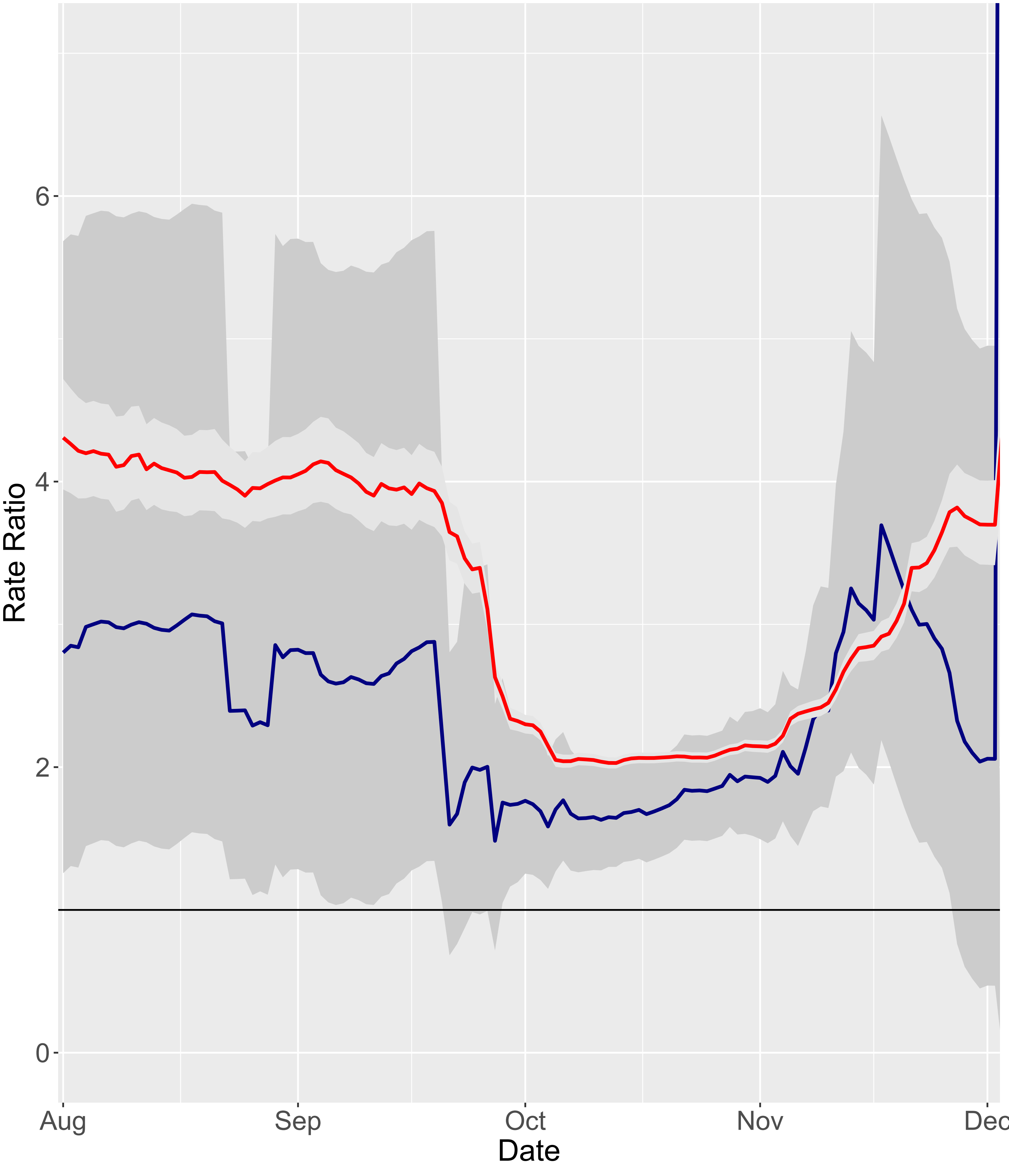

Supplement: Supplementary file 1 [file IRV-14-37-s001.pdf]
